# Evaluation of the quality of care of a multi-disciplinary risk factor assessment and management programme (RAMP) for diabetic patients

**DOI:** 10.1186/1471-2296-13-116

**Published:** 2012-12-05

**Authors:** Colman SC Fung, Weng Yee Chin, Daisy SK Dai, Ruby LP Kwok, Eva LH Tsui, Yuk Fai Wan, Wendy Wong, Carlos KH Wong, Daniel YT Fong, Cindy LK Lam

**Affiliations:** 1 Department of Family Medicine and Primary Care, The University of Hong Kong, 3/F Ap Lei Chau Clinic, 161 Main Street, Ap Lei Chau, Hong Kong; 2 Primary and Community Services Department, Hospital Authority Head Office, Hong Kong Hospital Authority, Hong Kong; 3 Statistics and Workforce Planning, Hospital Authority Head Office, Hong Kong Hospital Authority, Hong Kong; 4 School of Nursing, The University of Hong Kong, 4/F, William M. W. Mong Block 21 Sassoon Road, Pokfulam, Hong Kong

**Keywords:** Type 2 diabetes mellitus, Quality of life, Primary care, Prevention, Primary health care, Management programme, Risk prediction, Risk stratification

## Abstract

**Background:**

Type 2 Diabetes Mellitus (DM) is a common chronic disease associated with multiple clinical complications. Management guidelines have been established which recommend a risk-stratified approach to managing these patients in primary care. This study aims to evaluate the quality of care (QOC) and effectiveness of a multi-disciplinary risk assessment and management programme (RAMP) for type 2 diabetic patients attending government-funded primary care clinics in Hong Kong. The evaluation will be conducted using a structured and comprehensive evidence-based evaluation framework.

**Method/design:**

For evaluation of the quality of care, a longitudinal study will be conducted using the Action Learning and Audit Spiral methodologies to measure whether the pre-set target standards for criteria related to the structure and process of care are achieved. Each participating clinic will be invited to complete a Structure of Care Questionnaire evaluating pre-defined indicators which reflect the setting in which care is delivered, while process of care will be evaluated against the pre-defined indicators in the evaluation framework.

Effectiveness of the programme will be evaluated in terms of clinical outcomes, service utilization outcomes, and patient-reported outcomes. A cohort study will be conducted on all eligible diabetic patients who have enrolled into RAMP for more than one year to compare their clinical and public service utilization outcomes of RAMP participants and non-participants. Clinical outcome measures will include HbA1c, blood pressure (both systolic and diastolic), lipids (low-density lipoprotein cholesterol) and future cardiovascular diseases risk prediction; and public health service utilization rate will include general and specialist outpatient, emergency department attendances, and hospital admissions annually within 5 years. For patient-reported outcomes, a total of 550 participants and another 550 non-participants will be followed by telephone to monitor quality of life, patient enablement, global rating of change in health and private health service utilization at baseline, 6, 12, 36 and 60 months.

**Discussion:**

The quality of care and effectiveness of the RAMP in enhancing the health for patients with type 2 diabetes will be determined. Possible areas for quality enhancement will be identified and standards of good practice can be established. The information will be useful in guiding service planning and policy decision making.

## Background

Delivery of public-sector primary care in Hong Kong occurs through government-funded General Out-Patient Clinics (GOPC) managed by the Hospital Authority (HA). There are currently 74 clinics divided into seven geographical districts and referred to as HA clusters servicing the population of Hong Kong requiring government-subsidized health care. These primary care clinics provided 4,979,754 general outpatient attendances in 2010–2011 [1] and the number of attendances was estimated to increase further in the coming years. The majority of patients are elderly, of lower socio-economic status or have chronic diseases which require regular monitoring or medication such as hypertension and diabetes mellitus.

Type 2 diabetes mellitus (DM) is a major cause of morbidity and was the ninth commonest cause of death in Hong Kong in 2008 [2]. There are approximately 190,000 patients receiving care for DM in the GOPC according to data from HA. Until recently however, there have not been any formal standardized guidelines or protocols regarding the delivery of care for diabetic patients within the public primary care setting. In August 2009, the HA introduced a multi-disciplinary Risk Assessment and Management Programme (RAMP) to improve the quality of care for patients receiving diabetic care in the GOPCs. The RAMP utilizes a standardized protocol consisting of a workflow of checking of relevant clinical parameters including HbA1c, blood pressure (BP), low density lipoprotein-cholesterol (LDL-C), and an agreed risk assessment criteria for risk level stratification, with different management options assigned to patients of different risk levels and with different needs. Patients with DM who are independent in their activities of daily living and being followed up at regularly at the GOPCs are eligible to enter the RAMP. All enrolled patients undergo a comprehensive risk assessment and screening for diabetes-related complications, and are then assigned to receive appropriate interventions and education provided by a team of multi-disciplinary healthcare professionals according to their stratified risk level. Low risk patients continue with the usual GOPC follow up, medium risk patients are given additional intervention by an advanced practice nurse (APN), and high risk/very high risk patients are given additional intervention by an APN and an associate consultant, who is a specialist family physician. The RAMP is repeated once at least every one to two years for all patients who are enrolled.

A review of the international literature on risk stratification of diabetic patients supports the benefit of identifying high-risk patients through the clinical information system [3]. Risk factors can be identified based on the level of glycaemic control and/or presence of complications where improvement in HbA1c, BP, and LDL-C can be achieved through more intensive interventions [3,4]. Moreover, health service utilization such as Accidents and Emergency (A&E) department attendance, consultations, and hospital admissions can also be reduced [5,6]. A number of non-Asian countries have already successfully adopted such stratified models for chronic diabetic care including the United Kingdom, Australia, New Zealand and Canada [7-11].

In Asia, the Joint Asia Diabetes Evaluation (JADE) Program, incorporating a comprehensive risk engine, care protocol, clinical decision and self-management support has recently been developed to improve ambulatory diabetes care [12]. Although the JADE was developed based on data from patients receiving secondary or tertiary care, it forms the base for the stratification of local diabetic patients into very high risk, high risk, medium risk and low risk in the RAMP at primary care level. Apart from stratifying diabetic patients into different risk levels, equations integrating different patient’s clinical parameters have been formulated to predict the diabetic patient’s 5-year risk of coronary heart disease, stroke, end-stage renal disease, and all-cause mortality [12-15].

The goal of care for patients with DM is to prevent DM-related complications such as cardiovascular diseases and renal failure. Cardiovascular risk prediction rules have been developed, mostly based on the Framingham Study [16], but these may not be applicable to the Asian or Chinese population [17,18]. Studies have shown that the original Framingham functions might over-estimate the risk of coronary heart disease (CHD) in Chinese adults [18]. The Joint Asia Diabetes Evaluation (JADE) equations have been formulated and tested on local data from the Hong Kong Diabetes Registry showing good validity and discriminating power.

With the threat of aging population and the foreseeable increase in demand of public primary healthcare service on providing care to patients with chronic diseases, programmes that can be proved to be effective and provide a good quality of care are one of the solutions to deal with the complexity and demand of the health needs. Since DM is a common chronic condition with diverse complications in our locality as well as worldwide, we would like to evaluate the QOC and the effectiveness of the RAMP to prove if such kind of approach of chronic disease management works.

The evaluation of QOC and effectiveness is an essential step in assessing a chronic disease management programme on whether the intended care is provided and the expected health benefit is achieved. The information will influence future policy and service planning related to healthcare.

### Aims and objectives

The aim of this study is to evaluate the quality of care (QOC) and effectiveness of a multi-disciplinary risk assessment and management programme (RAMP) for type 2 diabetic patients attending government-funded primary care clinics in Hong Kong. The evaluation will be conducted using a structured and comprehensive evidence-based evaluation framework.

The objectives of the study are:

1) To review and identify the structure, process and outcome indicators of quality of care;

2) To identify the criterion and set the target standard for each indicator;

3) To compare the observed standards against the target standards;

4) To identify any on-site problems related to implantation of the RAMP;

5) To provide feedback on quality of care of RAMP;

6) To identify possible areas for improvement;

7) To give recommendations for enhancement of service delivery

The following hypotheses will be tested:

1) The structure and process criteria of care of different aspects should be achieved up to standards in all participating clinics;

2) A higher proportion of patients should have achieved the outcome targets for HbA1c, blood pressure, and LDL-C after the RAMP;

3) Patients participated in the RAMP should have better clinical outcomes than non-participants (control) managed by usual care;

4) Patients participated in the RAMP should not have higher health service utilization rates than non-participants (control) managed by usual care;

5) In longer terms, RAMP will lower the complication rate and cardiovascular risk level of diabetic patients.

## Methods/design

### Evaluation of quality of care

A longitudinal study using the Action Learning [19] and Audit Spiral methodologies [20] will be used to carry out a systematic analysis of the quality of care, and to identify areas for enhancement in a multi-disciplinary approach to chronic disease management. Audit is the best available tool to evaluate whether patients are receiving the best quality of care. It provides information to the service provider on how to improve the quality of health care delivery. It also reviews the procedures for assessment of patients, service delivery, outcomes and resources allocation. The audit can be regarded as a spiral systematic process, with the ultimate goal of improving the quality of care. The audit consists of different cycles and as the audit cycle continues, each cycle aims at a higher level of quality of care.

### Development of the evaluation framework

**Figure 1:**
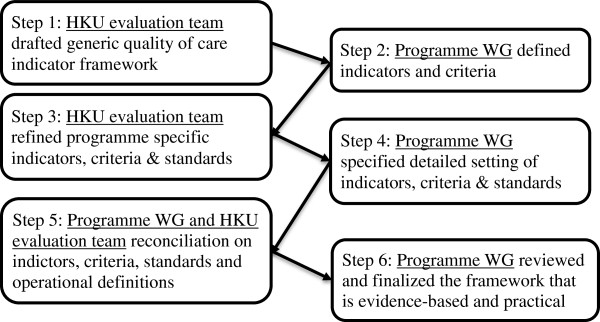
An iterative process and reconciliation between the HKU evaluation team and the programme working group (WG).

Donabedian’s taxonomy of quality of care on structure, processes and outcomes will be used as the evaluation framework [21]. A QOC framework (Additional file 1) was developed by an iterative process and reconciliation between the investigators and the programme providers (Figure 1). It started with the intensive literature review on DM so that key elements of management of DM at primary care level were grouped. Through iterative discussions with the working group (WG) of the health care providers (HA) and the statistical team of the HA on what aspects needed to be evaluated and what data are retrievable from the computer system, the evaluation framework gradually came into shape. This final evaluation framework listed out the indicators of the structure (staff, facilities, organization, and management), process (what, when and how care is delivered), and outcomes (clinical outcomes, service utilization and patient reported outcomes) with the required criteria and target standard of care to be achieved.

### Evaluation of the effectiveness of RAMP

Effectiveness of the programme will be evaluated in terms of clinical outcomes, service utilization outcomes, and patient-reported outcomes. A cohort study will be conducted on all eligible diabetic patients who have enrolled into RAMP for more than one year to compare their clinical and public service utilization outcomes of RAMP participants and non-participants. Clinical outcome measures will include HbA1c, blood pressure (both systolic and diastolic), lipids (low-density lipoprotein cholesterol); and the 5-yr and 10-yr cardiovascular risk or coronary heart disease risk (by using appropriate risk prediction equations). Public health service utilization rate will include general and specialist outpatient attendances, emergency department attendances, and hospital admissions. This cohort of patients will be followed up regularly and evaluated at assigned time-points, i.e. 6, 12, 36 and 60 months after the start of the study. For evaluation of the patient reported outcomes (PRO), 550 diabetic patients enrolled into RAMP and 550 diabetic non-RAMP patients will be recruited for telephone interview at the same time interval for their PEI, GRS, and private health service utilization rate.

### Subjects

All diabetic patients who have been enrolled into the RAMP will be included in the evaluation on process of care for each audit cycle. All enrolled subjects who have been recruited for more than 12 months in the programme will be included in the evaluation on the clinical outcomes of care for each audit cycle.

To identify the difference in programme performance and impact between the two groups (RAMP participants and non-participants), a cohort study approach would be adopted. The cohort includes all eligible diabetic patients under care of the public primary care outpatient clinics at 1 Sep 2009. The cohort would be evaluated annually on their clinical and service utilization outcomes from September 2009 to September 2015.

550 diabetic patients who have enrolled into the RAMP and another 550 diabetic patients who have not will be invited in person from the GOPCs by trained research assistants at the beginning of each audit cycle to take part in a telephone survey on PRO. All subjects who have signed the written consent to the telephone survey will be interviewed by telephone within four weeks, at 6, 12, 36 and 60 months.

### Sample size calculation

The sample size for the evaluation of quality of life change after RAMP is estimated to detect a minimally clinically important difference (MCID) in health-related quality of life (HRQOL) studies that is equivalent to Cohen’s small effect size of 0.3 [22]. A sample size of 350 patients in total (175 RAMP participants and 175 non-participants) is needed in order to have 80% power and 95% confidence interval by independent T-test [23]. Therefore taking into consideration these factors and allowing for a 25% dropout rate on each follow up, a total of 1100 patients (550 RAMP participants and 550 non-participants) need to be recruited.

### Data collection

#### Evaluation on structure and process

The coordinator of each of the seven clusters will be asked to complete a Structure of Care Questionnaire (Additional file 2). Anonymised data will be retrieved from the Hospital Authority’s patient information system to determine the patient recruitment rate, enrolment rate, risk level stratification, attendance at RAMP clinic, compliance with assessment as per protocol, number of investigations and referral rate. Information on patient and provider characteristics will also be collected as independent variables. The doctor-in-charge of the RAMP of each participating clinic will also complete the structure of care questionnaire (Additional file 2). We will ask them to submit a list of the staff and facilities designated for the programme, and a description of the programme objectives and protocol, and we will carry out site visits to cross-validate the data.

#### Evaluation on outcomes of care

Anonymised data on the relevant clinical outcomes, annual attendance rates for GOPC, specialist outpatient clinics (SOPC), A&E department and hospital admissions at baseline, 12, 24, 36 and 48 months will be retrieved from the Hospital Authority’s information system. The data will be extracted using specific data collection form. Data needed to calculate the future cardiovascular risk prediction (e.g. urine albumin to creatinine ratio) will be extracted from HA’s information system.

The 550 RAMP participants and 550 non-participants who have agreed to the telephone follow up will be contacted by a trained research assistant who will obtain written consent from each patient. Each patient will then answer the Chinese (Hong Kong) Short Form-12 version 2 (SF-12v2) Health Survey within four weeks from enrolment and a repeat survey at 6, 12, 36 and 60 months together with an assessment on patient enablement and global rating of change in health condition and a structured questionnaire on private health service utilization rates.

### Three audit and feedback cycles

An interim analysis of the standards of care, particularly on the structure and process of care was carried out 6 months after the start date of the evaluation study to identify major problems of programme implementation. These results were fed back and discussed with the programme team of HA to identify areas of deficiency and quality enhancement strategies.

**Figure 2:**
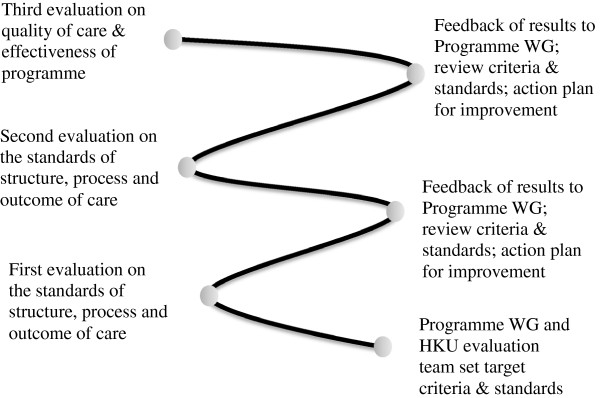
The audit spiral with three evaluation (audit) cycles.

Three evaluation (audit) cycles on the standards of care will be carried out at 15, 30 and 45 months from the start of the study to evaluate whether the set standards of care on structure, process and outcomes have been achieved (Figure 2). The first evaluation cycle is to identify gaps between practice and intended targets that may require changes in the structure and process of care. The second evaluation cycle is to determine the standards that are achievable after the programme has been established. The third evaluation cycle is to assess the sustainability of the standards of care. The results of each evaluation cycle on the performance of the programme will be fed back and discussed with the programme team of the Hospital Authority to identify any need for changes in quality criteria and standards and corresponding quality enhancement strategies to be implemented for RAMP.

### Outcome measures

#### Primary

1. The proportion of clinics that have satisfied the target of each of the structure criterion.

2. The number and proportion of diabetic patients who have completed RAMP.

3. The proportion of patients who have complied with the target of each of the process of care criterion.

4. The proportion of patients who have achieved the target of each of the outcome of care criterion, mainly the control of DM, as reflected by HbA1c.

5. DM-related complication rates, e.g. coronary heart disease, stroke, all-cause mortality, etc.

6. The predicted 5-year / 10-year cardiovascular risk, by using the risk prediction equations.

#### Secondary

1. The change in risk level of diabetic patients after RAMP

2. Other clinical outcomes, including SBP, DBP, LDL-C, and body mass index.

3. PRO measured by the change in SF-12v2 scores, the PEI and GRS scores at 6, 12, 36 and 60 months.

4. Service utilization outcomes measured by GOPC and SOPC attendance rates, A&E and hospital admission rates in the past 12 months.

### Study instruments

Study instruments will be used in evaluating the structure of care and the PRO. Evaluation of the process of care and outcomes do not involve use of study instruments as the necessary data is to be retrieved from the HA through the statistical team.

### Structure of care questionnaire

Structure of care questionnaire will be sent to the cluster co-ordinators and clinic doctors in-charge of the RAMP and require their input. The questionnaire covers questions on resources spent on RAMP, e.g. whether there is enough space for the programme, whether there is a data sharing platform within the programme, etc.

Three study instruments are used in the evaluation of the PRO, namely the short form-12 version 2 Health Survey, the Patient Enablement Instrument, and the Global Rating Scale.

A. The short form-12 version 2 (SF-12v2) Health survey

The Chinese (Hong Kong) SF-12v2 Health Survey will be used to measure HRQOL. It has been validated and normed on the general Chinese population in Hong Kong [24]. It measures eight domains of HRQOL on physical functioning, role physical, bodily pain, general health, vitality, social functioning, role emotional and mental health on a scale range from 0 to 100. A higher score indicates better HRQOL. The eight domain scores can be summarized into two summary scores, the physical (PCS) and mental (MCS) component summary.

B. The Patient Enablement Instrument (PEI)

The PEI is a measure of patient’s enablement in coping with the illness and life [25]. It has 6 items each rated on a 3-point (0, 1, and 2) scale. The summation of the item scores gives the PEI score with a higher score indicating better enablement. The PEI has been translated into Chinese and shown to be valid and reliable in the general Chinese population [26].

C. The Global Rating Scale (GRS)

The GRS is adapted from those used in studies by Jaeschke and Osoba et al. [27]. It assesses the subject’s global perception of any change in the overall health condition on a 7-point scale (−3, -2, -1, 0, 1, 2, and 3) over the last six months.

### Data analysis

Descriptive statistics on standard of care will be calculated by the percentage of clinics meeting the structure criteria, and percentage of subjects enrolled, attended, completed the programme, receiving criterion process of care, investigations and referral per protocol, and percentage of subjects achieving the criterion outcomes of care, in each audit cycle, and at the end of five years. Moreover, our primary analysis will use cardiovascular disease (CVD) risk-prediction algorithm according to the Framingham Heart Study [16]. This equation predicts the 10-year risk of CVD events which is defined as a composite of CHD (coronary death, myocardial infarction, coronary insufficiency, and angina), cerebrovascular events (including ischemic stroke, haemorrhagic stroke, and transient ischemic attack), peripheral artery disease (intermittent claudication), and heart failure. The equation is applicable to patients aged 30–74 years. As a means of cross-validation and comparison, the predicted 5-year cardiovascular risk will also be estimated by the locally validated JADE equations [12-15]. In addition, within-subject improvement in outcomes from baseline at 12, 24, 36 and 48 months will be analysed by paired t-test for continuous outcomes and McNemar test for binary outcomes. Furthermore, differences in outcomes between RAMP participants and non-participants, between audit cycles will be tested by analysis of variance (ANOVA) for continuous outcomes and Chi-square test for categorical outcomes. We will also compare the baseline frequency of attendance between RAMP participants and non-participants as a proxy measure of their motivation to care. Self-selection biases will be examined over inter-subject and inter-group differences by analysis of covariance. Adjustment for multiplicity where appropriate will be made by Bonferroni adjustment. At last, multiple regressions will be used to identify factors that are associated with quality of care or effectiveness of the RAMP.

### Ethics Approvals

This study has received ethics approval from the Institutional Review Board of the University of Hong Kong/ Hospital Authority Hong Kong West (UW 10–369), Hong Kong East (HKEC-2010-093), Kowloon East and Kowloon Central (KC/KE-10-0210/ER-3), Kowloon West (KW/EX/10-317 (34–04)), New Territories East (CRE-2010.543), and New Territories West clusters (NTWC/CREC/1091/12).

## Discussion

Quality assurance exercise on such a scale has never been conducted in Hong Kong, and only few have been conducted in Asia. This is the first territory wide study to evaluate the quality of care of a complex systematic intervention for diabetic patients in Hong Kong. The results can assure the quality and determine the effectiveness of this multi-disciplinary programme in improving the health and health-related outcomes for patients with DM. Possible areas for quality enhancement will be identified and standards of good practice can be established. Careful attention is required to facilitate coordination between the various parties. Action research requires the engagement of key stakeholders’ right from the beginning of protocol development and readiness to make changes in response to new issues that merged during the study process. Close collaboration with the programme administrator and providers are vital so that the results are valid and that recommendations made are feasible and acceptable.

### Coordination of stakeholder collaboration and communication

As the study involved health policy decision makers, Hospital Authority administrators and other staff from the Hospital Authority Head Office as well as Chiefs-of-service from seven regional clusters in addition to numerous frontline clinical staff, adequate and open channels for communication is essential for the smooth facilitation of the project. A senior project coordinator and single point of call representing the academic investigators and Hospital Authority teams helps to facilitate and streamline communication and arrangement of activities such as meetings, site visits, subject recruitment (for PRO) and monitoring of data collection.

Regular planning and feedback meetings are required to ensure all parties receive timely updates to the status of the evaluation and sharing of interim findings. The use of an iterative process is useful to facilitate stakeholder participation, however, careful planning is required to allow for efficient and effective two-way communication. Modifications to the evaluation framework, data collection and dissemination of results require endorsement by the decision-makers and the establishment of working groups is an effective way to manage the administrative and logistical challenges encountered when dealing with multiple parties.

### Feedback and planning meetings

Continuous communication and regular meetings with the working group, frontline staff, and statistical team is crucial in this continuous process of evaluation. Feedbacks and interim reporting help to identify deficiencies and direction of change at an earlier phase of the whole process of evaluation. Arrangement of site visit to the involved outpatient clinic and discussion with the frontline staff in addition to observation can help cross validate the accuracy of the data.

### Stakeholder endorsement and validation

In a programme of this scale, small variations in programme protocol are inevitable between sites. Every clinic needs to be able to have the flexibility to introduce their own refinements tailored specifically to accommodate the needs of their patient population. Other factors such as changes in availability of staff, space, and fluctuations in patient load can also contribute to variations in protocol delivery. To accommodate these variations, it is essential to verify with all participating clinics that the same operation skeleton is maintained and modifications from the protocol be minimised and maintained within an acceptable limits in order to totally reflect the performance and impact of the RAMP.

Furthermore, site visits to the clinics are an effective way to identify any potential on-site problems encountered in the actual implementation of the programme and suggest possible solutions.

### Development of the evaluation framework

The indicators and benchmark standards of care for the evaluation framework were derived from literature review and international and local management guidelines. However, research-based evidence is not always available for every criterion. In these circumstances, a compromise between best practice and local practice needs to be agreed upon with both policy decision makers and clinical healthcare providers. Furthermore, clear operation definitions are required to describe what is being measured, for example, explicit definitions for referral rates, enrolment rates and attendance rates need to be agreed to ensure that the information collected is accurate and interpretable.

Defining the target standards for each criterion of care is also essential and requires a fine balance between standards which are ideal and standards which are feasible. While the standard of care should be set appropriately high to allow room for improvement, it must still be realistic for use in real clinical settings.

### Operation definitions

In order to ensure the reliability of the responses of the Structure of Care Questionnaires, and to define clearly what exact information is needed and what exact data should be retrieved, operation definitions are set and supplemented to the evaluation reference framework so that the interpretation of the framework is standardized. Clear articulation, field testing, and modification of poorly understood items are required to avoid misinterpretation and assure accurate data collection, and it can be accomplished with the interim evaluation.

### Monitoring and ensuring data quality

Anonymised data on the process of care, clinical outcomes, and service utilization outcomes is extracted from the Hospital Authority’s information system. However, there may be substantial amount of missing data on clinical outcomes of care particularly for non-participants. It is because while one of the advantages of having RAMP is to ensure regular checking of patients’ clinical parameters like HbA1c, urine albumin-to-creatinine ratio, etc., patients who have not taken in RAMP (non-participants) are not secured to have these clinical outcomes checked regularly and their clinical data may not be as comprehensive as that for patients who have enrolled into RAMP (RAMP participants). Moreover, some information such as the duration of having DM of both RAMP participants and non-participants are self-reported which significantly influences the information accuracy. Therefore, every piece of information collected should be interpreted carefully in an attempt to truly reflect the performance and impact of the programme. To address these problems, it is important to discuss with the health policy decision makers, coordinators, and clinical staff whether it is feasible to collect the valid information as much as possible and the acceptable sampling period should be made. Thereupon, the operation definition for the information needed should be concluded (Additional file 1). For example, for the reading of HbA1c at baseline, the last available reading prior to the date of RAMP enrolment rather than those after should be adopted. The last HbA1c reading available in 9–15 months after RAMP enrolment should be used as the post-12 months reading.

## Conclusion

With the expected increasing numbers of patients with chronic diseases like DM, a well-organized and comprehensive multi-disciplinary approach to care will be the preferred model of healthcare delivery. Formal evaluation studies are needed to provide evidence that these models help to enhance the quality of care offered and received by the patients. If proven effective, similar multi-disciplinary models can be developed for management of patients with other chronic diseases like hypertension, chronic obstructive sleep apnoea, or osteoporosis at the primary care level. Studies looking to establish evidence for these models of care will be of considerable interest to health care planners, particularly when aging population is the global trend.

## Abbreviations

RAMP: Multi-disciplinary risk factor assessment and management programme (RAMP); DM: Diabetes mellitus; QOC: Quality of care; GOPCs: General outpatient clinics; BP: Blood pressure; LDL-C: Low density lipoprotein-cholesterol; APN: Advanced practice nurse; AC: Associate consultant; FM: Family medicine; A&E: Accidents and emergency; JADE: Joint asia diabetes evaluation; CHD: Coronary heart disease; SF-12v2: SF-12 health survey version 2.0; PEI: Patient enablement instrument; GRS: Global rating scale; MCID: Minimally clinically important difference; HRQOL: Health-related quality of life; SOPC: Specialist outpatient clinics; BMI: Body mass index; PRO: Patient reported outcomes; CVD: Cardiovascular disease; ANOVA: Analysis of variance.

## Competing interests

The authors declare that they have no competing interests.

## Authors’ contributions

CL initially conceived the study. All authors collectively designed and drafted the study protocol and sought funding and ethical approving. DF, CW, EW led on statistical analyses. CF, WW, CW, EW, DD, RK, ET, SW, CL contributed to recruitment and data collection. CF, WYC, CL provided assistance with drafting and management protocol. WW, CW were the project coordinator, assisted with recruitment and coordinated the data collection. CL, WYC, WW, CW, EW contributed to the drafting of the manuscript. CF is the PI of the funding application, coordinated the research network and research team, and drafted the manuscript. All authors have read the draft critically and approved the final manuscript.

## Pre-publication history

The pre-publication history for this paper can be accessed here:

http://www.biomedcentral.com/1471-2296/13/116/prepub

## Supplementary Material

Additional file 1 Quality of Care Evaluation Framework. Click here for file

Additional file 2 Structure of care questionnaire. Click here for file
