# Supplementary material for: Evaluation of the quality of care of a multi-disciplinary risk factor assessment and management programme (RAMP) for diabetic patients
Source: BMC Fam Pract. 2012 Dec 5;13:116. doi: 10.1186/1471-2296-13-116 (PMC3573901; doi:10.1186/1471-2296-13-116)
Supplement: Additional file 1 — Quality of Care Evaluation Framework. [file 1471-2296-13-116-S1.pdf]

Additional file 1: Quality of Care Evaluation Framework

Multi-disciplinary Risk Factor Assessment and Management Programme

Structure evaluation framework

Human Resources		Target Standard (% of teams)
1	There **must** be designated programme coordinator(s) to oversee the RAMP at cluster level.	100%
2	There **must** be a multidisciplinary team of healthcare personnel, including FM Specialist, Advanced Practice Nurse, Allied Health Professional to implement RAMP. Technical Service Assistant and General Service Assistant are required to support the operation.	100%
3	Allied Health Professionals (e.g. Dietitians, Podiatrists, Physio-therapists etc.) **should** be accessible to patients in the programme when indicated.	70%
4	RAMP team staff **must** be familiar with the programme objectives and logistics.	100%
5	RAMP team doctors and nurses **must** know the management protocol.	100%
6	RAMP team staff **should** have undergone relevant training for this programme.	70%
Office Infrastructure		Target Standard (% of teams)
7	Specific data collection forms (electronic) **must** be used for electronic documentation of patient data	100%
8	Professional staff of RAMP team **must** have access to the CMS system for patient data retrieval.	100%
9	There **should** be appropriate space provided for the programme.	70%
10	Facilities for patient recruitment, enrolment, assessment and RAMP sessions (e.g. intake / intervention sessions) **should** be available in the clinic.<br>List of essential facilities:<br>Fundus camera (per team)<br>Visual acuity charts<br>Monofilament and/or Biothesiometer<br>Access to laboratory service: a) renal function test, b) lipid profile, c) HbA1c, d) urine microalbumin	70%

11	Educational materials on disease knowledge **must** be available.	100%
Programme Management		Target Standard (% of clinics)
12	Patients enrolled in the programme **must** be properly documented in the CMS / OPAS.	100%
13	Patient enrolment records **should** be accessible to the doctors and other authorized members of the programme.	100%
14	The patient's doctor **should** be informed of patient's participation and RAMP classification.<br>*Being informed [i.e. Reminder/ record in the CMS/ patient hand-held record]*	70%
Organizational Structure		Target Standard (% of clinics)
15	There **should** be regular meetings among staff of each participating team to monitor the performance of the programme.	70%
16	There **should** be regular meetings between RAMP team staff and the cluster programme coordinator(s).	70%

Multi-disciplinary Risk Factor Assessment and Management Programme

Process evaluation framework

Service Delivery & Process of Care		Target Standard (% of patients, unless otherwise specified)
1	DM patients with or without HT **should** enroll in the RAMP.	50% (in the first year of operation)
2	Patients **must** be stratified into a RAMP risk group.	100%
3	Patients stratified into moderate risk group **could** be referred to nurse intervention session.	20%
4	Patients stratified into high / very high risk group **could** be referred to nurse intervention session.	30%
5	Patients stratified into very high risk group and with HbA1c > 8.4% **should** be referred to AC intervention sessions.	70%
6	Patients **could** be referred to patient empowerment programme (PEP). (*Only applicable to clusters with PEP*)	10%
7	Patients **could** be referred to the dietitian.	5%
8	Patients **could** be referred to the podiatrist.	5%
9	Patients who smoke **could** have at least 1 appointment at the smoking counseling and cessation centres (SCCC) after joining the programme. *Note: Patients with appointments in SCCC could be referred from any source, or they may have already attended SCCC before joining the programme.*	30%

Multi-disciplinary Risk Factor Assessment and Management Programme

Outcome evaluation framework

Clinical Outcomes		Target Standard (% of enrolled patients)
1	Patients **should** have HbA1c ≤ 7% one year after the programme.<br>*HbA1c reading one year right after the first intake assessment.*	35%
2	Patients **could** have improvement in HbA1c one year after the programme.<br>*Change in HbA1c before and one year after the first intake assessment.*	P < 0.05
3	Patients **should** have BP ≤ 130 / 80 mmHg one year after the programme.<br>*Average of two BP readings one year right after the first intake assessment.*	30%
4	Patients **could** have improvement in BP one year after the programme.<br>*Change in both SBP and DBP before and one year after the first intake assessment.*	P < 0.05
5	Patients **should** have LDL-C ≤ 2.6 mmol/L one year after the programme.<br>*LDL-C reading one year right after the first intake assessment.*	30%
6	Patients **could** have improvement in LDL-C one year after the programme.<br>*Mean change in LDL-C before and one year after the first intake assessment.*	P < 0.05
Service Outcomes (Secondary Outcomes):<br>12-month utilization of HA services before and after date of first appointment		Target Standard (% of patients)
7	Number of GOPC attendances **should** be decreased after the programme.	P < 0.05
8	Number of medical SOPC attendances **should** be decreased after the programme.	P < 0.05
9	Number of A&E attendances **should** be decreased after the programme.	P < 0.05
10	Number of hospital admissions **should** be decreased after the programme.	P < 0.05

Patient-Reported Outcomes		Target Standard (% of patients)
11	Patients **should** have improvement in quality of life (Short Form-12) 6 months after the programme.	P < 0.05
12	Patients **should** have global improvement in health (Global Rating Scale) 6 months after the programme.	70%
13	Patients **should** be more enabled (Patient Enablement Instrument) 6 months after the programme.	70%

Note:

All process and outcome indicators are confined to attending patients, unless otherwise specified.

**Descriptions**	**Definitions**
DM patients	Number of patients with GOPC attendances coded with ICPC-2 of 'T89' / 'T90', or with prescription of any pre-defined diabetic drugs in the reporting period. Patients attending medical SOPC with pre-defined diabetic drugs during the same period are excluded from analysis.
Enrolled patients	Patients with appointments for intake assessment.
Attending patients	Patients attended at least one session of the programme.

RAMP Structure Criteria – Operation Definition

	Data Required	Operation Definition
**Human Resources**		
1	There **must** be designated programme coordinator(s) to oversee the RAMP at cluster level.	Self-reported by Cluster Coordinator /Clinic Program Coordinator.
2	There **must** be a multidisciplinary team of healthcare personnel, including FM Specialist, Advanced Practice Nurse, Allied Health Professional to implement RAMP. Technical Service Assistant and General Service Assistant are required to support the operation.	Self-reported by Cluster Coordinator /Clinic Program Coordinator. (Please specify the number of programme staff and their time dedicated to the programme)
3	Allied Health Professionals (e.g. Dietitians, Podiatrists, Physio-therapists etc.) **should** be accessible to patients in the programme when indicated.	Self-reported by Cluster Coordinator /Clinic Program Coordinator. (Please specify the number of programme staff and their time dedicated to the programme)
4	RAMP team staff **must** be familiar with the programme objectives and logistics.	Self-reported by Cluster Coordinator /Clinic Program Coordinator. (Please attach a summary)
5	RAMP team doctors and nurses **must** know the management protocol.	Self-reported by Cluster Coordinator /Clinic Program Coordinator. (Please attach a summary)
6	RAMP team staff **should** have undergone relevant training for this programme.	Self-reported by Cluster Coordinator /Clinic Program Coordinator. (Please specify what training has been taken)
**Office Infrastructure**		
7	Specific data collection forms (electronic) **must** be used for electronic documentation of patient data.	Self-reported by Cluster Coordinator /Clinic Program Coordinator. (please enclose a print copy or print screen of all forms)
8	Professional staff of RAMP team **must** have access to the CMS system for patient data retrieval.	Self-reported by Cluster Coordinator/ Clinic Program Coordinator. (Please enclose a sample copy of relevant print screens please*)*
9	There **should** be appropriate space provided for the	Self-reported by Cluster Coordinator/ Clinic Program

	Data Required	Operation Definition
	programme.	Coordinator.
10	Facilities for patient recruitment, enrolment, assessment and RAMP sessions (e.g. intake/ intervention sessions) **should** be available in the clinic.	Self-reported by Cluster Coordinator/ Clinic Program Coordinator.
11	Educational materials on disease knowledge **must** be available.	Self-reported by Cluster Coordinator/ Clinic Program Coordinator.<br>(Please enclose a copy of relevant materials)
**Programme Management**		
12	Patients enrolled in the programme **must** be properly documented in the CMS / OPAS.	Confirm with HA support office record.<br>(Please enclose a sample copy of relevant print screens)
13	Patient enrolment records **should** be accessible to the doctors and other authorized members of the programme.	Confirm with HA support office record.
14	The patient's doctor **should** be informed of patient's participation and RAMP classification.	Confirm with HA support office record.
**Organizational Structure**		
15	There **should** be regular meetings among staff of each participating team to monitor the performance of the programme.	Self-reported by Cluster Coordinator/ Clinic Program Coordinator.<br>(Please specify the frequency and form of communication related to RAMP)
16	There **should** be regular meetings between RAMP team staff and the cluster programme coordinator(s).	Self-reported by Cluster Coordinator/ Clinic Program Coordinator.<br>(Please specify the frequency and form of communication related to RAMP)

RAMP Process Criteria – Operation Definition

Data Required	Operation Definition
**Service delivery**	
DM patients	Number of patients with GOPC attendances coded with ICPC-2 of 'T89' / 'T90', or with prescription of any pre-defined diabetic drugs in the reporting period. Patients attending medical SOPC with pre-defined diabetic drugs during the same period are excluded from analysis.
Enrolled Patients	Patients with appointments for intake assessment.
Attended Patients	Patients attended at least one session of the programme.
Completed Patients	Patients who have attended all sessions of the programme
**Process of Care**	
Patients identified with additional problems and requiring referral to other services	Patients identified with problems that require referral.
Referral to additional service	The number of patients referred for additional service to have their problem treated (Denominator: Patients identified to have problems)
Refusal of additional service	The number of patients who refused additional service to have their problem treated. (Denominator: Patients identified to have problems)

RAMP Outcomes Criteria– Operation Definition

Data Required	Unit	Operation Definition
**Clinical outcomes**		
HbA1c	%	The HbA1c reading from HA information systems at reference time points (baseline: last available before date of RAMP enrolment; post 12 months: the first reading in 9 - 15 months after RAMP enrolment)

Data Required	Unit	Operation Definition
Systolic BP	mmHg	The systolic BP readings from HA information systems at reference time points (baseline: last available before date of RAMP enrolment; post 12 months: the first reading in 9 - 15 months after RAMP enrolment).
Diastolic BP	mmHg	The diastolic BP readings from HA information systems at reference time points (baseline: last available before date of RAMP enrolment; post 12 months: the first reading in 9 - 15 months after RAMP enrolment)
LDL-C	mmol/L	The LDL-C reading from HA information systems at reference time point (baseline: last available before date of RAMP enrolment; post 12 months: the first reading in 9 - 15 months after RAMP enrolment).
RAMP risk level	-	Latest RAMP risk level<br>(1. Low risk; 2. Medium risk;3.High risk; 4.Very high risk
**Service utilization Outcomes (in the past 12 months)**		
Hospitalization	Episodes	Number of times the patient has been hospitalized in HA hospitals in the past 12 months
A&E attendance	Episodes	Number of times the patient attended HA A&E departments in the past 12 months
GOPC consultation	Episodes	Number of times the patient went for HA GOPC consultation in the past 12 months
SOPC medical consultation	Episodes	Number of times the patient went for HA SOPC (Medical) consultation in the past 12 months
**Patient reported outcomes**		
Health-related Quality of Life score	-	Measured by the SF 12-v2 domain scores, PCS and MCS scores
Patient Enablement Instrument (PEI) score	-	Measured by the mean PEI score and the proportion of subjects with PEI >0
Global Rating of Change Scale (GRS)	-	Measured by the mean GRS and the proportion of subjects with GRS score >0 or <0
