# Supplementary material for: Evaluation of the quality of care of a multi-disciplinary risk factor assessment and management programme (RAMP) for diabetic patients
Source: BMC Fam Pract. 2012 Dec 5;13:116. doi: 10.1186/1471-2296-13-116 (PMC3573901; doi:10.1186/1471-2296-13-116)
Supplement: Additional file 2 — Structure of care questionnaire. [file 1471-2296-13-116-S2.pdf]

Additional file 2: Structure of care questionnaire

Cluster Coordinator Questionnaire Number: ______________
Date: ______________

Multi-disciplinary Risk Factor Assessment & Management Programme (RAMP) Structure of Care Questionnaire

Please complete each question by circling your response choice or entering the appropriate information.

DRAFT

<table>
<tr><th colspan="5">Section A: Manpower and Human Resources</th></tr>
<tr><td>1</td><td>Is there a designated programme coordinator to oversee the RAMP at cluster level in your cluster?</td><td>1. Yes</td><td>2. No</td><td>3. Not sure</td></tr>
<tr><td>2</td><td>Is there a multidisciplinary team of healthcare personnel, including Family Medicine Specialist, Advanced Practice Nurse, Allied Health Professional to implement RAMP; as well as: Technical Service Assistant and General Service Assistant supporting the operation in your cluster?</td><td>1. Yes</td><td>2. No</td><td>3. Not sure</td></tr>
<tr><td colspan="5">Please indicate the total number following healthcare personnel and the percentage of their time dedicated to RAMP in all your cluster: (Put down ZERO if not applicable)

_____ full-time FM Specialist(s); each allocate _____% of their time.
_____ part-time FM Specialist(s); each allocate _____% of their time.
_____ full-time Advanced Practice Nurse(s); each allocate _____% of their time.
_____ part-time Advanced Practice Nurse(s); each allocate _____% of their time.
_____ full-time Allied Health Professional(s); each allocate _____% of their time.
_____ part-time Allied Health Professional(s); each allocate _____% of their time
_____ full-time Technical Service Assistant(s); each allocate _____% of their time.
_____ part-time Technical Service Assistant(s); each allocate _____% of their time.
_____ full-time General Service Assistant(s); each allocate _____% of their time.
_____ part-time General Service Assistant(s); each allocate _____% of their time.
Others:
____
____
____
____
__________</td></tr>
<tr><td>3</td><td>Are allied health professionals (Dietitian, Podiatrist, Physiotherapist and etc.) accessible to patients in your cluster when indicated?</td><td>1. Yes</td><td>2. No</td><td>3. Not sure</td></tr>
</table>

**Section B: Operational guidelines and protocols**				
4	Are you and your cluster RAMP team staff familiar with the objectives and logistics of RAMP?<br>("familiar": given an overview of the programme objectives and logistics. Please attach a summary)	1. Yes	2. No	3. Not sure
5	Do you and your cluster RAMP team doctors and nurses know the management protocol of RAMP?<br>("know": read and understood the programme management protocol. Please attach a summary)	1. Yes	2. No	3. Not sure
6	Have RAMP team staff in your cluster undergone relevant training for this programme?	1. Yes	2. No	3. Not sure
Please specify what training has been taken:<br>______________________________<br>______________________________				
**Section C: Programme Management and Organization**				
7	Are specific data collection forms (Metabolic Risk Assessment module)* used for documentation of patient data in your cluster?<br>(If yes, please enclose a sample copy or print screen of all forms)	1. Yes	2. No	3. Not sure
8	Do professional staff of your RAMP team(s) have access to the CMS system for data retrieval of patients in the RAMP of your cluster?<br>(Please enclose a sample copy of relevant print screens)	1. Yes	2. No	3. Not sure
9	Do the doctors and other authorized members of the RAMP in your cluster have access to the patient enrolment records (OPAS registration records)?	1. Yes	2. No	3. Not sure
**Section D: Premises and Facilities**				
10	Does your cluster have the following facilities provided for the RAMP?			
	a) fundus cameras?	1. Yes	2. No	3. Not sure
	b) visual acuity charts?	1. Yes	2. No	3. Not sure
	c) monofilaments to assess touch sensation?	1. Yes	2. No	3. Not sure
	d) biothesiometers?	1. Yes	2. No	3. Not sure
	e) laboratory services for the necessary investigations including renal function test, lipid profile, HbA1c and urine microalbumin?	1. Yes	2. No	3. Not sure
	f) appropriate space?	1. Yes	2. No	3. Not sure

DRAFT

<table>
<tr><td>11</td><td>Are educational materials on disease knowledge (type II diabetes mellitus) available in all the RAMP clinics in your cluster?
(Please enclose a copy of relevant materials)</td><td>1. Yes</td><td>2. No</td><td>3. Not sure</td></tr>
<tr><td>12</td><td>Are patients enrolled in RAMP in your cluster properly documented in the CMS/OPAS?
(Please enclose a sample copy of relevant print screens)</td><td>1. Yes</td><td>2. No</td><td>3. Not sure</td></tr>
<tr><td>13</td><td>Are the patients' doctors informed of their patients' participation in RAMP and their respective RAMP classifications?</td><td>1. Yes</td><td>2. No</td><td>3. Not sure</td></tr>
<tr><td colspan="5">If yes, please specify how:
____
____
______________________________</td></tr>
<tr><td colspan="5">**Section E: Governance**</td></tr>
<tr><td>14</td><td>Are there regular meetings among staff of each participating team in your cluster to monitor the performance of the RAMP?</td><td>1. Yes</td><td>2. No</td><td>3. Not sure</td></tr>
<tr><td colspan="5">Please specify the frequency and form of communication related to RAMP:
____
____
______________________________</td></tr>
<tr><td>15</td><td>Are there regular meetings between RAMP team staff, and the cluster programme coordinator in your cluster?</td><td>1. Yes</td><td>2. No</td><td>3. Not sure</td></tr>
<tr><td colspan="5">Please specify the frequency and form of communication related to RAMP:
____
____
______________________________</td></tr>
</table>

DRAFT

**Cluster characteristics**				
16	How many clinics are there in your cluster?	______________________		
17	How many clinics in your cluster participate in the RAMP?	______________________		
18	Does your cluster participate in:			
	Patient Empowerment Programme?	1. Yes	2. No	3. Not sure
	Shared care programme?	1. Yes	2. No	3. Not sure
	Nurse & Allied Health Clinics Programme?<br>______________________<br>______________________	1. Yes	2. No	3. Not sure

--- The End ---

DRAFT

Clinic Programme Coordinator Questionnaire Number: ______________
Date: ______________

Multi-disciplinary Risk Factor Assessment & Management Programme (RAMP) Structure of Care Questionnaire

This questionnaire should be completed by the person in charge of the programme in the clinic. One questionnaire should be completed for each clinic, i.e. the person in-charge needs to complete more than one questionnaire if he/she is in-charge of more than one clinic.

Please complete each question by circling your response choice or entering the appropriate information.

**Section A: Manpower and Human Resources**				
1	Is there a designated programme coordinator to oversee the RAMP in your clinic?	1. Yes	2. No	3. Not sure
2	Is there a multidisciplinary team of healthcare personnel, including at least one of each of the following: Family Medicine Specialist, Advanced Practice Nurse, Allied Health Professional to implement RAMP; as well as: Technical Service Assistant and General Service Assistant supporting the operation in your clinic?	1. Yes	2. No	3. Not sure
Please indicate the number of the following healthcare personnel and the percentage of their time dedicated to RAMP in your clinic: *(Put down ZERO if not applicable)*<br><br>_____ full-time FM Specialist(s); each allocate _____% of their time.<br>_____ part-time FM Specialist(s); each allocate _____% of their time.<br>_____ full-time Advanced Practice Nurse(s); each allocate _____% of their time.<br>_____ part-time Advanced Practice Nurse(s); each allocate _____% of their time.<br>_____ full-time Allied Health Professional(s); each allocate _____% of their time.<br>_____ part-time Allied Health Professional(s); each allocate _____% of their time.<br>_____ full-time Technical Service Assistant(s); each allocate _____% of their time.<br>_____ part-time Technical Service Assistant(s); each allocate _____% of their time.<br>_____ full-time General Service Assistant(s); each allocate _____% of their time.<br>_____ part-time General Service Assistant(s); each allocate _____% of their time.<br>Others:<br>______________________________<br>______________________________<br>______________________________				
3	Are allied health professionals (Dietitian, Podiatrist, and Physiotherapist and etc.) accessible to patients of your clinic when indicated?	1. Yes	2. No	3. Not sure

DRAFT

**Section B: Operational Guidelines and Protocols**				
4	Is your RAMP team staff familiar with the objectives and logistics of RAMP?<br>*(“familiar”: given an overview of the programme objectives and logistics. Please attach a summary)*	1. Yes	2. No	3. Not sure
5	Do your RAMP team doctors and nurses know the management protocol of RAMP?<br>*(“know”: read and understood the programme management protocol. Please attach a summary)*	1. Yes	2. No	3. Not sure
6	Has the RAMP team staff in your clinic undergone relevant training for this programme?	1. Yes	2. No	3. Not sure
Please specify what training has been taken:<br>______________________________<br>______________________________<br>______________________________				
**Section C: Programme Management & Organization**				
7	Are specific data collection forms (Metabolic Risk Assessment module)* used for documentation of patient data in your clinic?<br>*(If yes, please enclose a sample copy or print screen of all forms)*	1. Yes	2. No	3. Not sure
8	Do the professional staff of RAMP team have access to the CMS system for data retrieval of patients in the RAMP of your clinic?<br>*(Please enclose a sample copy of relevantprint screens)*	1. Yes	2. No	3. Not sure
9	Do the doctors and other authorized members of the RAMP in your clinic have access to the patient enrolment records (OPAS registration records)?	1. Yes	2. No	3. Not sure
**Section D: Premises and Facilities**				
10	Does your clinic have the following facilities provided for the RAMP:-			
	a) a fundus camera?	1. Yes	2. No	3. Not sure
	b) visual acuity charts?	1. Yes	2. No	3. Not sure
	c) monofilaments to assess touch sensation?	1. Yes	2. No	3. Not sure
	d) a biothesiometer?	1. Yes	2. No	3. Not sure
	e) laboratory service for the necessary investigations including renal function test, lipid profile, HbA1c and urine microalbumin?	1. Yes	2. No	3. Not sure
	f) appropriate space?	1. Yes	2. No	3. Not sure

DRAFT

11	Are educational materials on disease knowledge (type II diabetes mellitus) available in your clinic?<br>*(Please enclose a copy of relevant materials)*	1. Yes	2. No	3. Not sure	
12	Are patients enrolled to the RAMP in your clinic properly documented in the CMS/OPAS?<br>*(Please enclose a sample copy of relevant print screens)*	1. Yes	2. No	3. Not sure	
13	Are doctors in your clinic informed of their patients' participation in RAMP and their respective RAMP classification?	1. Yes	2. No	3. Not sure	
If yes, please specify how:<br>__________<br>__________<br>__________					
**Section E: Governance**					
14	Are there regular meetings among staff of each participating team to monitor the performance of the RAMP in your clinic?	1. Yes	2. No	3. Not sure	
Please specify the frequency and the form of communication related to RAMP in your clinic:<br>__________<br>__________<br>__________					
15	Are there regular meetings between RAMP team staff, and the cluster programme coordinator?	1. Yes	2. No	3. Not sure	
Please specify the frequency and the form of communication related to RAMP:<br>__________<br>__________<br>__________					
**Clinic Characteristics**					
16	Which cluster is your clinic in?	a. HKWC	b. HKEC	c. KWC	d. KCC
		e. KEC	f. NTWC	g. NTEC	
17	How many clinical staff do you have in your clinic?	a. Doctors ________	Nurses ________		
18	Is your clinic a family medicine training practice?	1. Yes	2. No	3. Not sure	
19	Does your clinic participate in:				
	a) Patient Empowerment Programme?	1. Yes	2. No	3. Not sure	
	b) Shared care programme?	1. Yes	2. No	3. Not sure	
	c) Nurse & Allied Health Clinics Programme?<br>__________<br>__________	1. Yes	2. No	3. Not sure	

--- The End ---

DRAFT
